# Is Human Chorionic Gonadotropin a Reliable Marker for Testicular Germ Cell Tumor? New Perspectives for a More Accurate Diagnosis

**DOI:** 10.3390/cancers17142409

**Published:** 2025-07-21

**Authors:** Nunzio Marroncelli, Giulia Ambrosini, Andrea Errico, Sara Vinco, Elisa Dalla Pozza, Giulia Cogo, Ilaria Cristanini, Filippo Migliorini, Nicola Zampieri, Ilaria Dando

**Affiliations:** 1 Department of Neurosciences, Biomedicine and Movement Sciences, University of Verona, 37134 Verona, Italy; nunzio.marroncelli@univr.it (N.M.); giulia.ambrosini@univr.it (G.A.); andrea.errico@univr.it (A.E.); sara.vinco_02@univr.it (S.V.); elisa.dallapozza@univr.it (E.D.P.); giulia.cogo_03@studenti.univr.it (G.C.); ilaria.cristanini@studenti.univr.it (I.C.); 2 UOC of Urology, Azienda Ospedaliera Universitaria Integrata di Verona, 37126 Verona, Italy; filippo.migliorini@aovr.veneto.it; 3 Department of Engineering and Innovation Medicine, Paediatric Fertility Lab, Woman and Child Hospital, Division of Pediatric Surgery, University of Verona, 37126 Verona, Italy; nicola.zampieri@aovr.veneto.it; 4 Saint Camillus International University of Health Sciencies (UniCamillus), 00131 Rome, Italy

**Keywords:** cancer biomarker, testicular germ cell tumors, male cancer, diagnostic marker, hormones, hCG

## Abstract

Testicular germ cell tumors (TGCTs) are the most common malignancies affecting young men between the ages of 14 and 44, accounting for about 95% of all testicular cancers. Despite being relatively rare compared to other cancers (~3.0 cases per 100,000 population, with high worldwide variability), TGCTs’ incidence is increasing, particularly in industrialized countries. The initial phase of TGCT diagnosis is performed by detecting in the blood the presence of three proteins, i.e., alpha-fetoprotein (AFP), lactate dehydrogenase (LDH), and human chorionic gonadotropin (hCG). Despite these proteins being defined as markers of TGCTs, they present limitations in specificity. Indeed, AFP is not elevated in pure seminomas; LDH serum levels can be elevated in other conditions, such as liver disease or tissue damage, and hCG can be elevated in both seminomas and non-seminomas, reducing its ability to differentiate between tumor types. However, the existence of hCG variants, characterized by distinct glycosylation profiles that are differentially expressed in TGCT types and subtypes, may increase the diagnostic and prognostic potential of this hormone. Furthermore, emerging molecular biomarkers, including miRNAs and tumor cells-related epigenetic status, may offer new promising alternatives to improve diagnostic accuracy. Nonetheless, standardized diagnostic protocols still need to be implemented. Finally, understanding the biological roles of hCG isoforms and their “canonical” (e.g., LHCGR) and “non-canonical” (e.g., TGF-βR) receptor interactions may help in understanding tumor biology and therapeutic targeting.

## Simple Summary

The most common solid malignancy affecting young males is testicular cancer, and almost 95% of the cases are germ cell tumors. They originate during embryogenesis, where primordial germ cells fail to differentiate, remaining in a quiescent state. Testicular tumors can manifest in children, adolescents, and adults under the influence of genetic, hormonal, and environmental factors, as well as gonadal disorders. The global incidence of testicular tumors is rising worldwide. Considering the negative impact that chemo and radiotherapies can have on fertility, it is important to enhance the specificity of serum tumor markers, streamlining early detection and facilitating prognostic monitoring. With this review, we aim to provide an overview of the potential roles that human chorionic gonadotropin (hCG) can have in testicular tumor progression, as well as its utility as a diagnostic and prognostic indicator when combined with already established and emerging serum tumor markers.

## 1. Introduction

### 1.1. Testicular Germ Cell Tumor

Testicular cancer is classified as a rare tumor according to the Global Cancer Observatory 2022 data, in which it ranks 27th in global cancer incidence [1]. Nevertheless, it is one of the most diagnosed tumors in males aged 14 to 44 years, accounting for approximately 74,000 new cases in men worldwide each year [2]. Testicular cancer exhibits a high incidence in Northern European countries, with rates up to 11.5 cases per 100,000 person-years, and significantly lower incidences in Africa, Asia, and South America, where the incidence can drop below 1 per 100,000 person-years [3]. However, in recent decades, there has been an increase in the incidence of testicular cancer in countries that were previously considered to have low rates of this disease [3,4]. TGCT is defined as one of the most treatable genitourinary cancers, thanks to the high efficiency of the current therapeutic approach, relying upon orchidectomy followed by surveillance and/or chemotherapy/radiotherapy administration based on risk factors and clinical stage [5]. However, survivors face an elevated risk of infertility, sexual dysfunctions, and other treatment-related side effects [4]. The estimated number of new testicular cancer cases in the United States for 2025 is approximately 10,000, with 600 attributable deaths for the same year, mainly related to those patients diagnosed at advanced stages [6]. The age-standardized rate (ASR) of testicular cancer per 100,000 man-years is projected to reach 9.98 by 2026, with higher rate increases in specific ethnic groups [7]. In Europe, the ASR of testicular cancer was 6.4 in 2022, as reported by the Global Cancer Observatory [8]. Overall, the total number of patients is projected to rise by 13% by 2035, despite population aging [9].

In approximately 90–95% of cases, testicular cancer originates from germ cells known as gonocytes, which are present in testicular cords during fetal and neonatal development [10] and initially develop into pre-invasive germ cell neoplasia in situ (GCNIS), leading to the final development of testicular germ cell tumor (TGCT) [11]. From a histological point of view, approximately 50–60% of TGCTs are classified as seminomas (SEs); the remaining 40–50% consist of non-seminomas (NSTs), while spermatocytic tumors account for less than 1% of all TGCTs [4]. SEs are usually diagnosed in patients between 25 and 55 years old, are considered homogeneous tumors presenting atypical gonocytes halted in the earliest stage of differentiation, and are usually characterized by slow growth. In contrast, NSTs are typically found in the age group of 15 to 35 years, are heterogeneous, and can manifest in various forms, including embryonal carcinoma, yolk sac tumor, teratoma, or choriocarcinoma [11]. NSTs are generally more aggressive precisely because of their more heterogeneous histological feature [12]; conversely, SEs typically exhibit a more indolent behavior, characterized by a generally better prognosis [12].

### 1.2. Mutations

TGCTs are characterized by distinct mutational landscapes (Table 1) that differ significantly from other malignancies with a low tumor mutation burden and high levels of aneuploidy. Notably, the gain of chromosome 12p, commonly manifesting as isochromosome 12p [i(12p)], is considered pathognomonic and the most common genetic hallmark for TGCT, being present in about 80% of patients [13]. Additional chromosomal changes include less frequent copies of 11q in SEs, and fewer copies of chromosomes 2q, 8, 9, 10q, 15, 19, and 22 in NSTs [12,14], thereby potentially contributing to TGCT histological subtype diagnosis.

Genetic predisposition, along with environmental and lifestyle factors, significantly contributes to the risk of developing familial TGCT, which is eightfold to tenfold more frequent among first-degree relatives of patients with TGCT [15]. A major reason for this increased occurrence is the enrichment of common single-nucleotide polymorphisms associated with higher TGCT risk in familial cases [16]. In this context, a recent meta-analysis of five genome-wide association studies highlighted that about 50 independent genetic markers correlate with 37% of the father-to-son familial risk [17]. Recurrent somatic mutations are relatively rare in TGCT [18], with the exception of the *KIT* oncogene, which is frequently mutated in SEs compared to NSTs [19], and the *RAS* oncogene, which is mutated in both SEs and NSTs [20]. The *KIT* gene encodes a stem cell growth factor receptor crucial for survival, proliferation, and germ cell migration [21]. SEs positive for *KIT* mutations show strong c-KIT activation at the biochemical level, playing a key role in the initiation and especially in tumor progression [22]. *KRAS* copy number gain and activating mutations initiate carcinogenesis by promoting cellular proliferation, gene expression, differentiation, mitosis, and cell survival [18]. *NRAS* is the second most commonly mutated GTPase in TGCTs, following *KRAS*, and supports the development of both pre-malignant and malignant germ cells. Lower frequency of the RAS-effector *BRAF* mutations has also been described in TGCTs, with higher incidence in chemo-resistant tumor forms [20]. Up to date, there are no approved targeted therapies aimed at inhibiting c-KIT and RAS/MAPK signaling pathways, even though some efforts are being made to develop tyrosine kinase inhibitors targeting c-KIT [23] and serine/tyrosine kinase inhibitors of RAS/RAF/MAPK signaling cascade to treat refractory or relapsed TGCT cases [24]. The presence of wild-type *TP53* is a common hallmark for TGCT, making it different from all other solid tumors and highly sensitive to cisplatin treatment, through the activation of extrinsic and intrinsic apoptotic pathways, including FAS death receptors, BAX, PUMA, and NOXA. However, mutually exclusive *TP53*/*MDM2* mutations/amplification have been described in a small subset of cisplatin-resistant or relapsed TGCT patients [25]. Moreover, the expression of wt *TP53* does not imply a correct functionality since post-translational modifications (PTMs) can inhibit its pro-apoptotic activity [26], and the presence of specific microRNAs, such as miRNA-372/373, can block p53 signaling [25]. Hence, this study and validation on cellular models of specific anti-miRNAs as new potential chemotherapy agents could implement the present therapy using a drug combination.

In addition to these genetic and post-translational factors, epigenetics plays a crucial role in TGCT development by regulating gene expression through DNA methylation, histone modifications, and non-coding RNAs [27]. Differential methylation patterns were found in SEs and NSTs. Most SEs have hypo-methylated genomes, with a small fraction showing hyper-methylation, which is a common feature of NSTs [28]. Since the chromatin state determines DNA accessibility to targeted therapies, the degree of methylation represents a crucial factor for tumor sensitivity to chemotherapy, enhancing the potential of epigenetic drugs as treatment options for TGCTs [29]. To date, several epigenetic drugs have been tested in vitro and in animal models as alternative treatment options for TGCT chemotherapies. These include the following: SGI-110 (DNA demethylating agent), which showed promising results in cisplatin-resistant TGCT; CBB3001 (histone demethylase inhibitor) able to arrest cell growth and downregulate pluripotency, while upregulating differentiation in TGCT cell lines; Romidepsin (histone deacetylase inhibitor) able to induce hyperacetylation leading to cell cycle arrest, apoptosis, and upregulation of stress markers in TGCT cell lines; and JQ1 (inhibitor of bromodomain and extraterminal domain) that leads to G1 growth arrest, apoptosis, and upregulation of stress markers [29].

Overall, genetic and epigenetic profiles of TGCT types and subtypes are not yet completely characterized and, in some cases, appear to have limited diagnostic significance. The need for standardization is fundamental to assess the distinctive distribution that may improve the diagnostic and prognostic value of classic serum tumor markers.

### 1.3. Metastatic Spread

TGCTs are very likely to metastasize, with around 37% of patients presenting with metastases at the time of initial diagnosis [30]. Common metastatic sites for TGCTs include the lungs, liver, retroperitoneal lymph nodes, central nervous system, and bones. One study conducted in a cohort of 969 patients, identified by using data from the Surveillance, Epidemiology, and End Results program (from 2010 to 2015), with a high metastatic burden and predominant NST histological markers, found that 90.5% of patients developed distal metastasis to the lungs; approximately 20% had liver metastasis, and 10% had both bone and brain metastases [31]. NSTs are more aggressive than SEs and are associated with a higher likelihood of visceral metastasis, including the lungs and liver. In contrast, SEs primarily metastasize to lymph nodes [32].

The current staging system for testicular cancer, known as Tumor, Node, Metastasis (TNM), classifies distant metastasis into different categories (Table 2), with non-pulmonary metastases indicating a more advanced disease and worse prognosis. Specifically, metastases to organs, such as the liver or bones, are considered more severe than those limited to the lungs or non-regional lymph nodes. This stratification is crucial for determining treatments and predicting outcomes [5]. Patients classified in the good prognostic group by the International Germ Cell Cancer Collaborative Group (IGCCCG) comprise 60% of those with metastatic germ cell tumors: among them, those patients having NSTs present a 5-year progression-free survival (PFS) rate of 90% and an overall survival (OS) rate of 96%, whereas patients with SEs present 89% and 95%, respectively [32]. However, poorer outcomes are often driven by extra-nodal metastatic involvement. Among those NST patients included in the poor outcome group by IGCCCG, the 5-year PSF drops to 54%, while the 5-year OS drops to 67% (Table 3) [32].

### 1.4. Diagnosis and Disease Management

Current approaches for identifying and treating testicular cancer focus on systematic diagnostic evaluations as outlined by the European Association of Urology (EAU) [5]. Physical examination is the first step in this process since the aforementioned malignancy commonly presents as a painless mass within the testicle. Some patients may report scrotal, abdominal, or back pain, indicating a delayed diagnosis. Therefore, the examination should also assess the abdomen, chest, and supraclavicular areas to evaluate disease progression effectively. Imaging plays a crucial role in testicular mass diagnosis, with high-frequency testicular ultrasound (>10 MHz) being the preferred method to differentiate between intra- and extra-testicular lesions. In this context, new ultrasound techniques are being developed to enhance malignant/benign lesion discrimination, such as contrast-enhanced ultrasound [33]. While scrotal magnetic resonance imaging (MRI) provides greater sensitivity and specificity than ultrasound, it is typically reserved for ambiguous cases or when considering testis-sparing surgery (TSS). For staging TGCTs, cross-sectional imaging of the thorax, abdomen, and pelvis is utilized as it is the most sensitive method, and is typically recommended before orchidectomy [5]. Following this surgical procedure, adjuvant therapies depend on clinical staging assessed by MRI or computed tomography (CT) scan. When these techniques are not sufficient in giving certain results in primary staging of TGCT, Positron Emission Tomography/Computed Tomography (PET/CT) is a useful tool to clarify disease status and to follow disease progression after chemotherapy, especially in testicular SE [34].

In addition to physical examination and imaging, a key point for TGCT diagnosis and management is the analysis of serum tumor markers, including alpha-fetoprotein (AFP), beta-human chorionic gonadotropin (hCGβ), and lactate dehydrogenase (LDH) [35]. The importance of these markers will be discussed in the next chapters. Following the diagnostic process, orchidectomy is the standard therapeutic approach for TGCT. In the case of benign, small, or indeterminate masses, negative tumor markers, and a normal contralateral testis, TSS may be considered to preserve fertility and hormonal function and may be accompanied by frozen section examination. After orchidectomy, the testis should be analyzed first from a macroscopic perspective, which includes assessing testis size, number of tumors present, and examining epididymis, spermatic cord, and tunica vaginalis. Second, they should be analyzed from a microscopic perspective, and a histological diagnosis should be performed to detect the presence or absence of lymphatic or peri-tumoral venous invasion [5].

Patients with GCNIS and normal contralateral testis can be managed by either orchidectomy or vigilant monitoring, as the five-year risk of developing TGCT is 50% [36]. In cases of solitary testis, local radiotherapy may be considered a part of the treatment plan. However, it can result in side effects, including reduced testosterone production and infertility [5]. Best immunohistochemical molecular markers to characterize and discriminate between GCNIS and benign lesions include OCT4, SALL4, PLAP, KIT, SOX17, D240, and NANOG [37].

In clinical stage (CS) I seminoma patients, orchidectomy alone is curative in 80% of cases and achieves almost 100% disease-free survival rates. Active surveillance, defined as a strict protocol of repeated cross-sectional imaging, monitoring STMs, and clinical assessment, is fundamental for the early identification of relapse that occurs in approximately 15% of patients [5]. Thus, it is fundamental to avoid unnecessary side effects given by adjuvant chemo and radiotherapies, which are recommended for patients at higher risks of tumor progression [38]. In this context, adjuvant chemotherapy involving carboplatin is effective with minimal long-term toxic effects; one cycle is usually sufficient. In contrast, adjuvant radiotherapy should be considered only for patients for whom chemotherapy is not a safe option.

For CS I NSTs, management strategies closely align with those for CS I SE. Retroperitoneal lymph node dissection (RPLND) has seen a reduction in its role due to higher cancer-specific survival rates of surveillance and lower relapse rates following adjuvant chemotherapy with one bleomycin, etoposide, and cisplatin (BEP) treatment cycle [5].

In cases of metastatic diseases, CS II SE patients typically undergo initial chemotherapy with three cycles of BEP or four cycles of etoposide and cisplatin (EP) if bleomycin cannot be used, with an overall survival of 99%. Alternatively, radiotherapy (2 Gy × 15 to a target dose of 30 Gy) can be administered. CS II NST patients with normal STMs, nerve-sparing RPLND is the recommended initial treatment, which can be followed by adjuvant chemotherapy to reduce the risk of relapse. In case of STMs positivity, patients should be treated with chemotherapy (BEP 3× or EP 4×) [5].

For CS IIC and III SE and NST patients within the worst prognostic risk groups, BEP 4× or four cycles of EP or, alternatively, a combination of etoposide, ifosfamide, and cisplatin (VIP) should be administered [5]. Even though most of the patients will be cured with a five-year relative survival rate of approximately 95%, in Western Europe, it is important to take into account that these therapies have detrimental effects on the fertile potential of these patients, who, as mentioned, are generally affected by testis cancer at an age that could fit with the longing for fatherhood.

## 2. Cryptorchidism as a Crucial Risk Factor for TGCT

### 2.1. Cryptorchidism

Cryptorchidism is a primary form of testicular dysfunction characterized by the failure of the testes to physiologically descend from an intra-abdominal position into the scrotum. It is the most frequent congenital birth defect in male children and can occur as both a standalone disorder or in association with other congenital abnormalities (syndromic cryptorchidism). Approximately 4% of all newborn males have at least one undescended testis [39]. Although about half of these cases spontaneously descend during the first three months of life, almost 1% of all males remain cryptorchid at the end of their first year. Cryptorchidism occurs bilaterally in one-third of the cases and unilaterally in two-thirds of cases, with a frequency of 1–5% in full-term newborns and 30% in premature newborns [40]. Cryptorchid testes are classified as ectopic or high/low abdominal, inguinal, supra scrotal, and high scrotal based on their position along the usual descent route. In the clinical setting, however, a simple distinction between palpable and nonpalpable and between unilateral and bilateral is mostly used [40].

Although cryptorchidism is often considered a mild malformation, it represents the best-characterized risk factor for infertility and testicular cancer in adulthood. The relative risk of developing TGCT in individuals with a history of cryptorchidism is 4 to 7.5 times higher than that of the general population [41,42]. Moreover, these individuals are frequently subfertile in adulthood due to spermatogenic impairment, which is most commonly observed in bilateral cases. Abnormal sperm parameters are observed in about 80% of men with a history of bilateral cryptorchidism and in about 30% of those with unilateral cryptorchidism. Although the association between cryptorchidism and TGCT has been clinically established, the mechanisms leading to carcinogenesis are still unknown [43]. The lack of reliable animal models hinders research efforts. Thus, there is an urgent need for ongoing investigation into germ cell development and the oncogenic mechanisms associated with cryptorchidism.

### 2.2. Surgical Treatment

Cryptorchidism treatment has evolved considerably over the years. In Europe, since the first half of the 20th century, the most advocated and widely used therapeutic approach for testicular descent failure was the administration of hormonal therapy, given the belief that gonadotropin and androgen deficiency were one of the etiological factors underlying the causes of undescended testicles [44]. Thus, the most used therapy consisted of the intramuscular injection of hCG for its ability to mimic the effects of luteinizing hormone (LH) in Leydig cells. Despite testosterone levels significantly increasing after administration, the treatment success rate was only 19% [45]. In addition to the poor efficacy, hCG injections in 75% of the patients showed adverse effects, such as increased hairiness and greater aggressiveness, which could diminish after treatment arrest [46]. For these reasons, the 2016 European Urology guideline does not recommend hormonal treatment to induce testicular descent in cryptorchid patients, and to date, the surgical approach, called orchidopexy, is the treatment of choice in 95% of cases, recommended between 6 and 12 months of age, and with high success rates [47]. Orchidopexy has been shown to conserve spermatogenesis [40], support hormone production, and decrease the risk of testicular tumors or other future complications [39]. There are several inguinal and scrotal orchidopexy techniques, each of which is designed to address specific anatomical and clinical situations associated with cryptorchidism. Following surgery, the testicle brought to the scrotal site is often smaller in size and has a reduced blood supply compared to the contralateral testicle. Promising clinical data are showing that adjuvant hCG therapy appears to help in restoring the operated testis’s functionality and size by potentially improving its vascularity, volume, and morphology [48,49].

### 2.3. Testicular Alterations of Cryptorchid Testicles

Despite the performance of cryptorchidism surgical correction via orchidopexy, testicular function frequently remains suboptimal, highlighting the need for long-term follow-up, including regular evaluation of endocrine function and fertility potential. Indeed, cryptorchidism can lead to long-term consequences on testicular function, such as disrupted spermatogenesis and increased risk of testicular cancer, likely due to innate abnormalities inherent within the testicular tissue [50]. The neoplastic risk is elevated by four to six times in undescended testicles, whereas it is less than twice as high in descended ones. The risk for cancerous development escalates with the height of the testis alongside its descent route [51]. Among the various possible locations of undescended testes, those located within the abdomen present the highest risk of developing testicular cancer. Approximately 10% of all testicular tumors originate from cryptorchid testes, with the intra-abdominal positions accounting for the highest malignancy rate, about 5%, and particularly involving seminomas, which is the predominant tumor type in these cases [51,52]. Cryptorchid patients generally present with low testicular volume, germinative epithelial degeneration, apoptosis, vacuolation, and impairment of Sertoli cells [53], which are the orchestrators of spermatogenesis, supporting fetal germ cell commitment and development [54]. Germ cell maturation starts with gonocytes differentiating into spermatogonia, usually completed by 6 months of age. A key determinant in the maturation process is a transient but essential period of hormonal activation occurring in early infancy known as “mini-puberty.” This phase is characterized by a surge in LH, follicle-stimulating hormone (FSH), and testosterone, which plays a critical role in driving the transformation of gonocytes into adult dark (Ad) spermatogonia. At 3 months, Ad spermatogonia differentiate and proliferate, but these events are reduced in cryptorchid boys, indicating a failure in gametogenic cell maturation. This reduction in spermatogonia is more severe than the reduction in the total number of germ cells. The unequal reduction in Ad spermatogonia is directly related to future spermiogram outcomes and represents a fundamental abnormality in germ cell development in cryptorchidism [55]. Furthermore, sperm DNA damage significantly increases in cryptorchid subjects, and sperm density is variable in both unilateral and bilateral cryptorchid males. Most of the previous studies that focused on semen quality have used the World Health Organization (WHO) criteria of ≥16 × 10^6^ spermatozoa/mL as the lowest normal sperm concentration [56], highlighting a better sperm quality in persistent unilateral cryptorchidism than in bilateral one [57]. Finally, over the years, up to the most recent studies suggest that the patient’s age, their level of sexual maturity, and the degree of germ cell damage are important considerations in achieving restoration of germ cells and normal sperm production [53].

## 3. TGCT Serum Diagnostic Markers

The fact that the average age at which TGCT is diagnosed involves young patients represents a double-edged sword: on the one hand, good health and young age allow patients to tolerate even very intensive therapies, ensuring complete eradication of the tumor; however, on the other hand, the age of tumor onset usually coincides with the period of life in which a man would like to become a father, but the therapies have the disadvantage of undermining the reproductive capacity and fertile potential. Prevention, therefore, becomes a crucial point for this type of cancer in order to intercept the development of a new tumor formation from the beginning and, therefore, improve the life expectancy of patients under the various aspects previously listed. As reported above, there are standard serum tumor markers indicated in the EAU guidelines [5], i.e., AFP, LDH, and hCG [58,59]. However, recently, specialists have questioned their actual usefulness, suggesting that they may be insufficiently specific or indicative. In this sense, the limitations of the aforementioned markers have been demonstrated [60]. However, many meta-analysis papers aimed to identify new markers that may be more sensitive and specific for TGCT have been published, leading to a future improvement in their clinical use [61,62,63].

### 3.1. Alpha-Fetoprotein

AFP is produced by the fetal liver, whose concentration is quite high at birth and tends to decrease rapidly thereafter [64]. The levels of this protein could also increase during life due to lesions affecting the liver, rare diseases, ataxic telangiectasia, hereditary tyrosinemia [65], and some tumor pathologies. Among neoplasms that involve an increase in the production of AFP [66], there are hepatocellular carcinoma and cancers involving the stomach, pancreas, biliary tract, and lungs, in which AFP can be used as a tumor marker to support their diagnosis. Regarding testis cancer, AFP has been demonstrated to be secreted by NSTs (primarily by yolk sac tumor and, to a lesser extent, by embryonal carcinomas) and not by SEs; therefore, patients with an elevated protein level should present a non-seminomatous component of testis cancer, opening the way to the improvement in the specificity of AFP detection in the sera of patients with NSTs. In this regard, a recent work has reported a correlation between the expression ratio of the main TGCT serum markers, including AFP, compared to the volume of the tumor mass, to verify the relationship of their expression with the survival of patients without recurrence. The authors demonstrated that patients with nonseminomatous tumors present high AFP levels at diagnosis, together with a poor survival prospect [59].

Even though biomarkers are included in international surveillance guidelines, high-quality evidence on their accuracy, optimal thresholds, and the most effective surveillance strategies using modern investigative techniques remains limited [60], especially for AFP levels in pure seminomas.

### 3.2. Lactate Dehydrogenase

LDH is an enzyme found in nearly all living cells. It catalyzes the reversible conversion of pyruvate to lactate while oxidizing NADH to NAD^+^. An increased amount of LDH in the blood may be a sign of tissue damage and some types of cancer or other diseases, along with being an indicator of normal tissue turnover. In mammals, there are three types of LDH isoenzymes, namely, LDHA, LDHB, and LDHC, which form homo- or hetero-tetramers with different tissue specificities and catalytic efficacies [67]. Given that serum LDH level is frequently elevated in cancer patients and is linked to poor clinical outcomes and therapy resistance, its measurement is best suited as a prognostic marker and for tracking cancer development before and after therapies. Nevertheless, it is well known that it is not a tumor-specific protein and, thus, cannot help in identifying the type of tumor from which the patient may be affected. To this important consideration must be added that tests aimed to verify the presence of the enzyme are based on the analysis of the activity and not on its total quantity, with the consequent effect that in each laboratory, different values are obtained based on the type of assay performed, making the reference ranges extremely variable [68]. Related to this last point, it should be emphasized that LDH activity is influenced by the hemolysis of red blood cells in the taken sample, which, by releasing their own protein, causes false positive values that can be further altered by normal cellular necrosis. Unfortunately, all those listed are molecular obstacles that create major limitations to the clinical utility of LDH as a marker [69].

Although it is a generic marker indicating various forms of tumor, in the specific case of testicular cancer, LDH is generally considered a non-specific marker associated with tumor burden and metastasis in both SEs and NSTs [59]. However, due to its versatility, it is still used to detect and monitor a variety of medical conditions, quickly giving a prompt response to doctors to understand the patient’s condition [70].

### 3.3. Human Chorionic Gonadotropin

HCG is a hormone produced by the placenta during pregnancy. Its levels rise after conception and continue to increase until about 10 weeks into pregnancy [71]. Some cancerous tumors produce this hormone; therefore, elevated levels measured when there is no pregnancy may lead to a cancer diagnosis and, if high enough, paraneoplastic syndromes [72]. HCG is a heterodimeric glycoprotein composed of an α (alpha) subunit (chromosome 6q14.3) identical to that of LH, FSH, and thyroid-stimulating hormone (TSH), and a β (beta) subunit (chromosome 19q13.3) that is unique to hCG, and which is, therefore, what is looked for in hematological tests.

HCG is highly glycosylated; indeed, almost 30% of its molecular weight is made up of sugar side chains, allowing for the existence of different glycosylation statuses. In this context, five distinct molecules have been described: three dimer variants consisting of the “canonical” hCG, hyperglycosylated hCG (hCG-H), and sulfated hCG, which all share the same aminoacidic structure but different carbohydrate compositions, and two hCGβ monomeric variants, one of which is hyperglycosylated (hCGβ-H) [73,74,75].

Notably, during early pregnancy, cytotrophoblasts produce hCG-H, which is believed to function as an autocrine factor by signaling through the TGF-β receptor (Tβ-RII), thus enhancing the invasive capabilities of extravillous cytotrophoblasts [76]. The same hCG-H isoform, together with hCGβ and hCGβ-H, can be detected in serum and urine of NST patients diagnosed with a choriocarcinoma subtype component, thus supporting pathological angiogenesis and tumor invasion, with no or low LHCGR involvement [73,76,77]. Other NST subtypes that have been demonstrated to express and secrete hCG-H and hCGβ include embryonal carcinoma and mixed germ cell tumors [78]. Conversely, in SEs whose composition comprises multinucleated syncytiotrophoblasts (approximately 30%), hCGβ and lower amounts of hCG can be detected [78,79]. This hormone is also used as a postoperative marker, preceding the search for radiological evidence of the disease; indeed, following hCG half-life kinetics in patients who have had orchidectomy provides staging and prognostic information [80], being able to assess whether residual disease is present, with persistent or rising levels suggesting active tumor cells and possible relapse. Nevertheless, it is important to be aware that despite hCG being the marker with the highest positive predictive value of TGCT relapses, false-positive events can still occur [81]. In detail, the scenarios of cases in which a patient can encounter false-positive results can be varied and caused by different factors. For example, the use of specific narcotics alters hCG tests: cocaine triggers proteins similar to those produced during pregnancy; marijuana interferes with hormone levels, and heroin mimics certain hormones, such as hCG. Then, some individuals may have circulating factors in their serum that interact with the hCG antibody, and the most common are heterophilic antibodies. Finally, most of these false-positive results are due to the detection of pituitary human chorionic gonadotropin [82].

Nowadays, it is well known and established that one of the characteristic traits of cancer conditions is a good vascularization of the tumor microenvironment to allow tumor cells to receive sufficient nutrients to proliferate; they themselves can increase vascularization in their favor, and this has also been demonstrated in the case of testicular cancer [83]. Specifically, it has been highlighted how hCG has a key role in stimulating angiogenesis [49]. In this way, a correlation has been found between high levels of hCG in the blood of patients with testicular cancer and their poor prognosis [83]. Although it seems, to date, the marker on which diagnoses can be based most reliably, there are still some aspects that need to be explored and explained, especially at the molecular level, represented by the presence of different isoforms and the lack of standardized diagnostic kits clearly able to differentiate them. Moreover, the clinical challenges represented by false positives and the absence of hCG expression by some TGCT histological subtypes underscore the urgent need to develop new molecular markers and to implement the current marker detection.

### 3.4. MicroRNAs as New Potential Diagnostic Markers

MicroRNA (miRNAs) are single-stranded molecules consisting of 19–22 nucleotides, which function as post-transcriptional regulators thanks to their specific sequences and recognition patterns, thereby modulating gene expression [84]. Due to their resistance to RNAse degradation, high stability in body fluids, tissue-specific expression patterns, and expression alterations during testicular cancer transformation, miRNAs are potential TGCT diagnostic and prognostic biomarkers [85]. Many studies show how miRNA expression varies between malignant and benign tissues and among different tumor types. In recent years, specific miRNA clusters have been selected as potential diagnostic and prognostic markers for TGCTs. Notably, the focus has centered on miRNA-371a-3p and miRNA-375 [86]. Primarily, miRNA-371a-3p is known to be mainly overexpressed in SEs, mixed NSTs, and embryonic carcinoma, usually combined with the presence of wild-type p53, and exploiting its oncogenic activity by inhibiting *LATS2* (large tumor suppressor kinase 2) [87]. It is considered the most sensitive (90%) and specific (>90%) molecular serum marker for TGCTs, whose expression permits the follow-up of tumor burden after each chemotherapeutic cycle and identifies the presence of viable germ cell tumor components in residual masses with almost 100% sensitivity [88]. Significantly, this biomarker is presently being integrated into clinical trials and mentioned in EAU guidelines due to promising results about its capabilities to outperform traditional serum tumor markers, such as AFP, hCGβ, and LDH, in most TGCT subtypes, with a notable exception being teratoma, in which miR-371a-3p expression is generally absent [5].

Conversely, miRNA-375 presence has been reported in other NST subtypes, such as teratomas, yolk sac tumors, and mixed tumors containing those two [12], and is also being investigated as a possible marker for identifying scar tissue in residual masses ≥ 1 cm following chemotherapy for stage II tumors [12]. However, several studies found no association between miRNA-375 serum levels and teratoma presence [88,89,90,91]; thus, current miRNA technology seems not to be able to overcome clinical challenges in teratoma diagnosis and management. Other preliminary studies investigated miRNA clusters comprising the miRNA-302/367, which shows oncogenic activity by targeting a tumor suppressor gene upstream *SPRY4* (a regulator of MAPK/ERK and PI3K/Akt pathways), mainly in the embryonal carcinoma subtype [87]; miRNA-223-3p, which is supposed to trigger its oncogenic action by inhibiting FBXW7 activity (one of the crucial components of the tumor suppressor SCF-ubiquitin-ligase complex) [87]; and miRNA-21,-29a, and -106b clusters that seem to be a promising tool for early detection and post-chemotherapy follow-up in TGCT patients [92].

In conclusion, miRNAs can be a useful tool in both diagnosis and post-chemotherapy residual mass management in TGCTs, but they still have some limitations. The main one is the lack of a standardized approach for miRNA detection and evaluation, and the setting up of cutoff values may be a challenge, considering the high variability in miRNA expression among different TGCT subtypes. Nevertheless, multiple ongoing clinical trials aim to address existing issues in the miRNA clinical utility. Notably, the clinical trial NCT03385655 is focused on examining the application of serum miR-371a-3p as a biomarker for monitoring disease in patients with TGCT, whereas NCT04435756 aims to evaluate its efficacy in identifying viable tumors within residual masses following chemotherapy. These investigations are expected to validate miRNA assays, set standardized cutoff values, and assess the cost-effectiveness of miRNA-guided management strategies, potentially leading to more personalized and less invasive care for TGCT patients [93,94].

### 3.5. Other Potential Markers

Recent investigations have explored innovative TGCT biomarkers pertinent to risk evaluation, screening, diagnostic procedures, prognostic assessments, predictive analysis, and recurrence monitoring. Among these, cell-free DNAs (cfDNAs) are DNA fragments found in human blood plasma due to apoptosis/necrosis or active secretion in response to both physiological and pathological manifestations. Several studies demonstrated the alteration of cfDNA expression in TGCT patients’ blood samples [95,96,97,98,99]. Overall, cfDNA as a biomarker for TGCT shows a sensitivity of about 50–80%, which is higher than serum tumor markers but lower compared to miRNAs, and a specificity of 89–100%, which is equal to serum tumor markers and higher than that of miRNA [100]. One interesting point is the possibility of combining miRNA-371a-3p and *RASSF1A* cfDNA methylation status to detect teratoma patients, as this TGCT subtype is usually difficult to detect with classical serum tumor markers and miRNA alone [98].

Despite the rapid increase in cfDNA interest among several cancer types, including lung, breast, and colorectal cancers, research on TGCT is far behind. The reason for that may be found in the overall low amounts of detectable cfDNA in patients’ serum, the high variability of cfDNA concentration among healthy controls (0–100 ng/mL) and TGCT patients (5–1000 ng/mL), and the lack of standardized pre-analytical and analytical methods [100].

Another potential TGCT marker may be represented by circulating tumor cells (CTCs), cells released from the primary tumors and transported through blood or lymphatic vessels, that have also been demonstrated to play a role in metastasis in solid tumors. A study identified CTCs in 11.5% to 17.5% of patients with germ cell tumors, with a higher frequency observed in metastatic cases [101]. Critical limitation in CTCs detection is hindered by protein markers used to recognize them, which exhibit low sensitivity, below 60%; furthermore, CTCs do not consistently correlate with tumor burden [102]. Molecular evaluation of gene expression can help discriminate between histological TGCT subgroups. In this context, one study performed an in silico and histopathological analysis at the DNA, RNA, and protein levels to implement new prospective biomarkers (HOXA9, MGMT, CFC1, PRSS21, RASSF1A, and MAGEC2) with already used ones, including c-KIT, OCT3/4, Sall4, and PLAP [61]. The resulting data obtained by combining bioinformatic analyses of DNA epigenetic modifications and mRNA expression with histopathological protein assessment showed significant differences among TGCT subtypes. However, further analyses are needed to corroborate these data.

Finally, another study investigated promising predictive markers of survival and therapeutic response by profiling the mRNA expression of *GRK4*, *PCYT2*, and *RGSL1* in TGCT patients [103].

## 4. hCG as a Critical Marker for TGCT Diagnosis

### 4.1. hCG Levels During TGCT Developmental Stages

HCG, as well as AFP and LDH, as reported above, represent clinically used serum tumor markers of TGCT for primary diagnosis, staging, monitoring of therapeutic response, and follow-up [104,105]. Overall, as already mentioned, serum levels of the tumor markers (S) have low sensitivity in identifying TGCT, AFP, and/or hCG serum levels are elevated in 73% of NSTs, while AFP is never elevated, and hCG is elevated in approximately 30% of SE cases [79].

According to the 2016 TNM classification of the International Union Against Cancer (UICC) for TGCT, hCG levels in the blood are classified as staging serum tumor markers, as follows: not available or normal value in Sx or S0, less than 5000 mIU/mL in S1, from 5 to 50,000 mIU/mL in S2, and greater than 50,000 mIU/mL in S3. This range, together with the characteristics of the primary tumor, the involvement of the lymph nodes, and the presence of metastases, allows for the staging of the TGCT in stage S0, stage IS, stage II A-C, and stage IIIA-C (Table 2): (i) stage S0 refers to GCNIS that presents abnormal cellular formation localized in the seminiferous tubules, without spreading in other parts of the testicle; in this case, the hCG reference levels are not analyzed and information not collected; (ii) stage I refers to the earliest stage of testicular cancer that is only present in the testicle, without spreading; hCG is not detectable in the blood in stage IA and stage IB, but is detectable in stage IC, in which there is evidence of vascular or lymphatic invasion, and at least one of the tumor markers levels is raised, with hCG levels less than 5000 mIU/mL; (iii) stage II refers to cancer cells that have spread from the testicle into nearby lymph nodes; stage II is split into stage IIA, IIB, and IIC, depending on how many lymph nodes are nicked by cancer cells and their size; β-hCG levels are included in the range 5000–50,000 mIU/mL; (iv) stage III refers to a cancer that has spread to lymph nodes or other organs, with β-hCG levels higher than 50,000 mIU/mL [5].

The IGCCG classification system lists the metastasized NSTs in good, intermediate, or poor prognosis according to the staging of the hCG serum marker. In particular, given the presence of testis/retroperitoneal primary tumor and the absence of non-pulmonary visceral metastases, S1 or S2 serum levels allow for the classification in the good or intermediate prognosis group. Fifty-six and 28% of the cases of NSTs are present in the first and second groups, respectively. The remaining 16% of the cases are in the poor prognosis group characterized by primary mediastinal, non-pulmonary visceral metastases, and S3. This classification system directly impacts the choice of therapy to be performed (Table 3).

Serum marker levels should be assessed before and after orchidectomy, since AFP and hCGβ have half-lives of 5–7 and 1–3 days, respectively. In addition, the persistence or the increase in tumor marker levels after orchidectomy usually indicates metastatic disease. Exceptions may be those rare patients with hypogonadism, liver disease, and hereditary AFP elevation, who have an elevated hCGβ or AFP value without TGCT, which may be considered false positives [106].

To monitor the chemotherapeutic response, serum markers are evaluated on the day of initiation of the first treatment cycle, every week if they are elevated, otherwise, at the beginning of the new therapy cycle. After the end of chemotherapy, a biochemical and radiological reassessment should be performed [104]. Only 40–50% of patients who experience relapse and undergo active surveillance for clinical stage I disease have an increase in serum markers; the same circumstance happens for 30% of patients who receive chemotherapy. Note that about 10% of all patients with recurrence have only increased serum markers with no evidence of metastatic disease by imaging [107].

Lastly, from a molecular point of view, Arrieta et al. correlated the increased serum hCG levels with vascularization in TGCT. In particular, patients with hCG serum levels ≥ 25 mIU/mL had an increased tumoral vascular neoformation, suggesting that hCG could be involved in angiogenic processes during tumor development [83].

### 4.2. hCG Expression and Epigenetic Regulations

Among the two subunits of hCG, the serum levels of the β subunit strictly correlate with tumor burden, indicating poor prognosis if highly concentrated [68], whereas the levels of the α subunit seem to be consistently produced in excess. Indeed, the limiting factor of the total amount of secreted hCG is linked to the expression of the hCGβ subunit [108], which is regulated by intricate epigenetic mechanisms, including DNA methylation, histone modifications, and transcription factor interactions.

**Figure 1:**
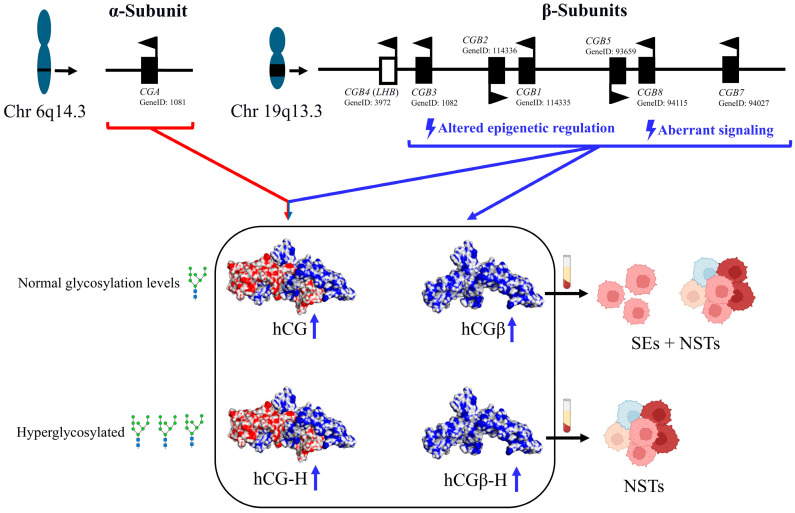
Schematic representation of the hCG α-subunit gene locus (*CGA*, chromosome 6q14.3) and the β-subunits gene cluster (*CGB* genes, chromosome 19q13.3), and the four possible hCG isoforms (represented by the 3D model P0DN86-1) detectable in the serum of specific TGCT patients: normally glycosylated hCG and hCGβ, and their hyperglycosylated counterparts (hCG-H and hCGβ-H). The presence and relative abundance of these isoforms vary between SEs and NST subtypes.

HCGβ is encoded by six tandemly arranged allelic genes (*CGB1*, *CGB2*, *CGB3*, *CGB5*, *CGB7*, and *CGB8*) likely generated from an ancestral *LHB* (*CGB4*) gene by duplication events throughout evolution (Figure 1) [109]. *CGB1* and *CGB2*, initially considered to be pseudogenes, are indeed transcriptionally active but with unknown biological activity [110]. *CGB7* is classified as a type I gene, whereas *CGB3*, *CGB5*, and *CGB8* are categorized as type II genes. The primary structural distinction between these two groups lies in the variation in only three amino acids.

An uneven gene expression pattern has been recently observed in the *CGB* gene cluster among healthy and tumorigenic tissue. For example, in ovarian cancer, the increased expression of hCGβ is associated with demethylation of *CGB* promoter regions. Furthermore, the expression levels of type II genes strongly correlate with the transcription factor AP-2 alpha (TFAP2A) levels [111]. In bladder cancer patients, high expression of hCGβ type II genes is an indicator of high-grade disease. Determining the type II to type I ratio in urine could be a potential method for diagnosis and follow-up [112]. Analysis of *CGB* gene expression in TGCT could uncover unique expression patterns. Indeed, NSTs produce hCG-H alongside the free β subunits, while SEs primarily express hCGβ (Figure 1). The presence of hyperglycosylated hCGβ isoforms raises questions about the capabilities of hCGβ detection kits to correctly quantify such isoforms, potentially reducing both sensitivity and specificity. Overall, hCG levels can be a significant predictor of relapse in TGCT patients [113].

The regulatory elements of all *CGB* genes contain binding sites for various transcription factors, many of which are the same across these genes. In this context, both basal and cAMP-induced expression are regulated by transcription factors, such as TFAP2A, a selective transcription factor SP1 and SP3, octamer-binding transcription factor OCT3/4, PPARγ, p53, and metastasis-associated protein 3 (MTA3) [114,115,116,117,118]. Although the presence and activity of transcription factors are essential for gene expression regulation, they do not completely account for the differences in expression levels of individual genes in both normal and cancerous tissues. This indicates that additional mechanisms, such as DNA methylation and histone modification, can play a fundamental role in controlling the accessibility of DNA regulatory sequences. Aberrant de novo epigenetic changes can lead to abnormal gene expression, which is crucial in cancer development. However, analyzing the methylation status of individual *CGB* genes is challenging due to their nearly identical sequences, close chromosomal proximity, and the gene cluster’s tendency for gene conversion [119]. As for miRNA detection, the lack of a standardized approach is another major limitation in translating epigenetic findings into clinical practice. This challenge, combined with highly cellular heterogeneity, especially in mixed NSTs, makes result interpretation particularly difficult. Furthermore, the relationship between epigenetics and *CGB* gene expression has primarily been investigated in normal placental tissue, characterized by higher degrees of hypomethylation. This association has also served as the basis for numerous studies focused on pregnancy failure, ovarian cancer, and breast tumors, highlighting a specific demethylation pattern leading to the overexpression of certain *CGB* variants [111].

### 4.3. Role of Hormones in hCG Expression

HCG is a pluripotent hormone that regulates ovulation, implantation, pregnancy maintenance, and fetal development. Regulatory mechanisms of this hormone involve complex physiological and pathological mechanisms, implying autocrine and paracrine signaling pathways and various cellular and molecular interactions. Its biological activity is specific to the β-subunit and not limited to the interaction with LHCGR. Indeed, interactions between hCG-H and transforming growth factor beta receptor (TGFβR) have been reported [120]. Physiologically, hCG is released by the placental syncytiotrophoblastic cells, and it stimulates the *corpus luteum* to synthesize progesterone to maintain the pregnancy. Lower amounts of hCG are also produced in the pituitary gland, liver, and colon. Pathologically, several hCG variants can be expressed in serum, urine, or tumor tissue by placental or other trophoblastic tumors, as well as non-trophoblastic tumors, such as germ cell tumors [121].

Expression of hCG genes is regulated by several hormones (gonadotropin-releasing hormone (GnRH), prolactin, progesterone), growth factors (vascular endothelial growth factor (VEGF), epidermal growth factors (EGF)), cytokines (Interleukin (IL)-6 and -1, tumor necrosis factor (TNF)-α), ligands of the nuclear receptor PPARγ, and the homeobox gene (*DLX3*) [120].

GnRH is a secretory decapeptide playing fundamental roles in mammalian reproduction. Although the hypothalamus and pituitary gland are the primary sources and targets of GnRH, it is also found in extra-hypothalamic tissues, including the placenta and testes, where both GnRH and GnRH receptor (GnRH-R) are present [122]. Placental GnRH acts as a paracrine regulator of hCG secretion from the trophoblast during pregnancy. Testicular GnRH is believed to be expressed by Sertoli cells, whereas GnRH-R was identified at the protein level in testicular interstitial cells, including Leydig cells. It has been shown that mRNA transcript levels of both GnRH and GnRH-R are significantly elevated in men with spermatogenic failure [123]. Additionally, GnRH may function as an autocrine or paracrine regulator in the development of tumors in reproductive tissue [122].

Prolactin (PRL) is a pleiotropic hormone fundamental for mammal lactation regulation. It is released from lactotrophic cells of the anterior pituitary as well as extra pituitary cells. In females, PRL regulates the hypothalamic–pituitary–ovarian axis, thus influencing ovarian follicle maturation and ovulation [124]. In males, hyperprolactinemia contributes to infertility in approximately 11% of oligospermic individuals by inhibiting the pulsatile secretion of the GnRH. This suppression leads to a decreased pulsatile secretion of FSH, LH, and testosterone, ultimately resulting in spermatogenic arrest, impaired sperm motility, and altered sperm quality. Moreover, hyperprolactinemia directly affects spermatogenesis and steroidogenesis by acting on prolactin receptors found in Sertoli cells and Leydig cells within the testes, which can lead to primary hypogonadism and infertility [125]. Although limited data exist on the effects of PRL on hCG synthesis and secretion, one study reported correlations between hCG synthesis and PRL exposure time in placental primary cell cultures [126].

Progestational steroids and their immediate metabolites can suppress hCG secretion in vitro. Specifically, physiological concentrations of progesterone have been shown to suppress hCG secretion, which may explain the decline in maternal hCG levels during pregnancy. This suppression of hCG by progesterone in the syncytiotrophoblasts is comparable to how steroids modulate pituitary gonadotropins within the hypothalamic–pituitary axis [127]. However, there is no information about the possible effects of progesterone on TGCTs.

In summary, hCG secretion regulation is a complex process influenced by autocrine and paracrine mechanisms, various cellular signaling pathways, and multiple external hormonal factors. The hormone-dependent regulation of hCG variant expression by cancer cells within the tumor microenvironment remains largely unknown. Exploring this area could uncover potential therapeutic targets for disrupting proliferative and anti-apoptotic signals in cancer cells, thereby providing new opportunities for treatment strategies.

### 4.4. hCG Receptor: LHCGR and Its Variants

Luteinizing hormone/chorionic gonadotropin receptor (LHCGR) is a member of the class A guanine nucleotide-binding protein-coupled receptors (GPCRs) and belongs to the subfamily of glycoprotein hormone receptors (GPHRs). LHCGR plays a critical role in regulating fetal development and in the establishment of primary and secondary sex characteristics. It is primarily expressed in females within ovarian theca, granulosa, and luteal cells, whereas in males, it is predominantly found in testicular Leydig cells. The overall organization of the *LHCGR* gene is highly conserved across species. In humans, the gene locus is located on the short arm of chromosome 2 (2p21), consisting of 11 exons and 10 introns. In addition to this conserved structure, humans and other primates possess an extra exon known as exon 6A, resulting in a total of 12 exons and 11 introns [128]. Notably, an additional splice variant has been reported in the common marmoset monkey (*Callithrix jacchus*), where the wild-type form of LHCGR completely lacks exon 10 [129].

The LHCGR physiological importance in sexual development and reproduction is underscored by the phenotypic manifestations of activating and inactivating receptor variants. At least 84 *LHCGR*-damaging variants have been documented [130]. In males, partial or total receptor inactivation can lead to type I or II Leydig cell hypoplasia (LCH). Conversely, increased sensitivity of LHCGR to its ligands or constitutive activation can result in familial or sporadic male-limited precocious puberty, a condition that can be related to a higher risk of developing TGCTs when a germline pathogenic LHCGR variant is present [131]. At the same time, somatic gain-of-function variants have been associated with Leydig cell adenomas [132].

In females, most LHCGR variants exhibit minimal effects on reproductive function; however, some inactivating polymorphisms are linked to conditions such as oligo-/amenorrhea [133], infertility [134], empty follicle syndrome [135], and higher risks of developing polycystic ovarian syndrome [136]. Activating variants, such as p.18insLQ, have been reported to correlate with shorter disease-free survival in breast cancer patients [137].

These receptor function and expression variations highlight the complexity of *LHCGR* regulation and its consequences on reproductive health. A particularly interesting aspect of this regulation is the presence of exon 6A, which leads to at least three different LHCGR mRNA transcripts that can vary in ratio under pathological conditions [138]. The primary transcript results in a full-length receptor containing exons 1–11 and composed of 699 amino acids, with a molecular weight of 85–95 kDa when mature and fully glycosylated [139]. Exon 6A can be spliced as either a terminal or internal exon; however, the internal variant contains a premature stop codon that prevents its translation, while the terminal variant (exons 1–6A) produces a truncated protein containing 209 amino acids (theoretical mass of 25 kDa) that is highly expressed, at levels comparable to the full-length LHCGR transcript [140]. The functional role of this variant remains unclear. Kossak et al. first described the exon 6A variant and demonstrated a strong correlation between mutations in this cryptic exon and human diseases, including Leydig cell hypoplasia and male pseudohermaphroditism [138,140]. This mutation altered the relative amounts of LHCGR splice variants, leading to an accumulation of internal variant transcripts that are degraded due to nonsense-mediated decay, resulting in insufficient functional receptor expression on cell membranes. Given these alterations in receptor expression, it is important to explore the functional implications of the LHCGR exon 6A terminal variant. The ability of this variant to bind hormones is not fully characterized; however, sequence alignment suggests strong similarities between this variant and the full-length receptor, indicating that it may theoretically bind LH and hCG through its eight leucine-rich repeats, potentially functioning as a hormone scavenger [139]. Moreover, the presence of various LHCGR isoforms in certain GCNIS and seminomas has been recently described in the literature and confirmed to be present at both transcriptional and translational levels [141]. Direct comparison between primer sets targeting extracellular and intracellular LHCGR regions highlighted the possible presence of truncated and full-length variants, detectable in seminomas and some GCNIS specimens, while NSTs showed low or undetectable LHCGR expression. This expression pattern was confirmed at the protein level, where LHCGR has been detected both as the canonical transmembrane receptor and as a secreted lower molecular weight protein. The presence of a functional LHCGR receptor on the surface of seminoma cells opens the possibility of a direct stimulatory effect on tumor cells upon hCG stimulation, confirmed by both extensive literature data and the presence of sporadic GCNIS in patients harboring activating LHCGR mutations [141]. Additionally, the presence of biologically unknown circulating LHCGR variants (possibly the one including exon 6A) can support the usage of this receptor as a diagnostic/prognostic marker in TGCT patients. However, LHCGR does not seem to have the diagnostic potential to differentiate TGCTs from benign conditions. Nevertheless, elevated serum LHCGR levels in patients with larger seminomas suggest a possible prognostic marker. Finally, the fact that LHCGR expression is higher only in seminoma patients with elevated LDH serum marker further corroborates the connection with tumor burden [141].

## 5. Improvement in an Effective Diagnosis of TGCT Based on hCG Expression and Signaling

**Figure 2:**
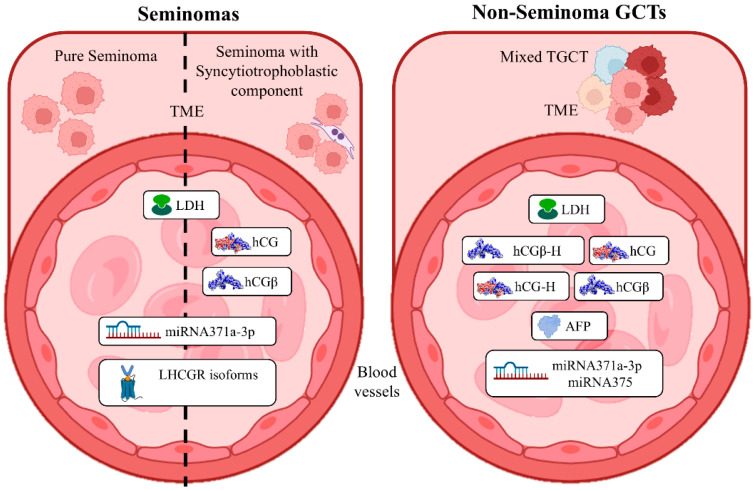
A schematic overview of the distribution of established serum tumor markers, including AFP, LDH, and hCG, alongside emerging biomarkers such as miRNAs, LHCGR variants, and hCG isoforms in patients with SE and NSTs. On the left, seminomas are divided into pure and those containing a syncytiotrophoblastic component, shown within the tumor microenvironment (TME). The main difference in biomarkers among these two SE subtypes is the lack of hCG and its free β-subunit in the pure seminoma. On the right, mixed TGCTs, which include two or more TGCT subtypes, exhibit a broader biomarker profile, in accordance with their histological complexity. These include LDH, AFP, both normally glycosylated and hyperglycosylated hCG and hCGβ, as well as miRNA371a-3p and miRNA375.

Early cancer detection is an increasingly pressing need, especially when the resulting therapy involves gonadotoxic treatments, as in the case of testicular tumors, which lead to radical damage to germ cells, resulting in infertility. Moreover, the predictive identification of patients with a high risk of developing malignancy as early as the pediatric age allows for timely disease management through targeted follow-ups, specific evaluations, and preservation of their fertile potential. Starting from this comes the need to identify targeted and unequivocal diagnostic markers (Figure 2). In addition, if this aspect is combined with an easy biochemical evaluation, for example, through blood or urine analysis, these markers would be easily applicable to improve risk stratification and surveillance in high-risk groups (e.g., cryptorchidism or family history), serving as a routine screening tool for testicular tumors.

Considering the biological significance of the three serum markers used in the clinic to diagnose and monitor TGCT, hCG could be the most clinically impactful hormonal marker, although it is not produced and secreted in all TGCT patients. In this context, it is necessary to classify TGCT tumors based on their incidence, developmental stage, prognostic group, and the typical markers expressed, among which we focused our attention on hCG (Table 4). Regarding testicular cancer in general, the classification based on staging serum tumor markers analyzed before chemotherapy can be divided into different groups, as extensively discussed in Section 4.1. Despite this classification defining clear parameters, it is important to highlight that there is an evident heterogeneity of hCG isoforms and expression among the different TGCT subtypes, as reported in Table 4.

**Table 4:** Summary of the main TGCT types and subtypes with clinical statistics and treatment strategies.

**Germinoma Family of Tumors (~50%)**							
**ICD-O-3.2 subtypes**	**Terminology**		**Incidence**		**Median Age**	**hCG, hCGβ marker positivity**	**References**
9061/3	Seminoma		~70%		30–49 years	Negative	[35,78,142,143]
9061/3	Seminoma with syncytiotrophoblastic cells		~30%			Positive	
**Prognosis group**	**5-year PFS**	**5-year survival rate**	**Clinical stages**		**Treatment** **(Orchidectomy mandatory first step for all CSs)**		**References**
Good	89%	95%	I		Surveillance; adjuvant carboplatin or radiotherapy		[5]
			IIA/B		Chemotherapy (BEP × 3 or EP × 4) or radiotherapy (30–36 Gy)		
			IIC and III		Chemotherapy (BEP × 3 or EP × 4)		
Intermediate	79%	88%	IIC and III		Chemotherapy (BEP × 4 or VIP × 4)		
Poor	Nd	Nd	Not classified		Nd		
**Non-seminomatous germ cell tumors (~50%)**							
**ICD-O-3.2 subtypes**	**Terminology**		**Incidence pure**	**Incidence mixed**	**Median Age**	**hCG, hCG-H, hCGβ, hCGβ-H marker positivity**	**References**
9070/3	Embryonal carcinoma		16%	80–90%	30 years	Positive	[144,145,146,147,148]
9071/3	Yolk sac tumor		<1%	44%	16–20 months	Rarely positive	
9100/3	Choriocarcinoma		<1%	8%	25–30 years	Strongly positive	
9080/3	Teratoma (post-pubertal)		4–9%	50%	20–35 years	Slightly positive in mixed forms	
**Prognosis group**	**5-year PFS**	**5-year survival rate**	**Clinical stages**		**Treatment** **(Orchidectomy mandatory first step for all CSs)**		**References**
Good	90%	96%	I		Surveillance, adjuvant chemotherapy (BEP × 1), and primary RPLND		[5]
			IIA/B		Nerve sparing RPLND, adjuvant chemotherapy		
			IIC and III		Chemotherapy (BEP × 3 or EP × 4)		
Intermediate	78%	89%	IIC and III		Chemotherapy (BEP × 3 or EP × 4)		
			IIA/B		Chemotherapy (BEP × 4 or VIP × 4)		
Poor	54%	67%	IIC and III		Chemotherapy (BEP × 4 or VIP × 4)		

Therefore, it is important to specify that during diagnostic or prognostic serum tumor marker analysis in patients suspected of having TGCT, international guidelines on testicular cancer do not distinguish among all hCG variants. To our knowledge, most immunoassay kits used to monitor hCG levels in patients’ serum rely upon identifying both hCG (standard dimeric form) and hCGβ subunit, excluding their hyperglycosylated forms.

Despite that, considering together both NSTs and SEs, hCG and/or hCGβ are still the most reliable among the three serum markers, with a sensitivity of 35%. Specifically, for NST patients, the sensitivity of hCG and AFP is similar, around 42%, and for SE patients, hCGβ and LDH present the highest sensitivities of 30% [102]. Detecting hyperglycosylated hCG and hCGβ variants, combined with standard serum tumor markers, could significantly increase their diagnostic and prognostic potential.

Concerning the majority of SE tumors that do not contain a syncytiotrophoblastic component (~80%) [35,78,142,143], diagnosis and follow-up based on the evaluation of only AFP and LDH serum levels are not sufficient, as they are generally not elevated in this kind of testicular tumor type [149]. Moreover, NSTs without a trophoblastic component, i.e., spermatocytic seminomas, pure teratomas, and yolk sac tumors, do not show any hCG immunoreactivity [78]. Clearly, this high variability in hCG expression and glycosylation pattern, together with situational low sensitivity of LDH and AFP, makes the diagnosis more complicated. To address these limitations, miRNA-371a-3p and miRNA-375 are emerging as promising TGCT biomarkers. Furthermore, a deep study of the cellular composition of the testes of cryptorchid patients would shed light on this developmental impairment, helping to understand whether the predisposition of these young patients to develop TGCT is intrinsic or could be addressed with specific therapeutic approaches.

Other important aspects worth considering are the signaling events triggered by hCG. First, hCG binds to the same receptor as LH, i.e., LHCGR, the stimulation of which activates diverse signal cascades in support of cell growth and proliferation. Thus, the expression of this receptor could be another helpful marker that can be investigated in the serum or urine of TGCT patients, especially by considering that truncated variants of LHGCR, as described above, are supposed to be soluble and to sequester LH and hCG from the stream. For these reasons, it is reasonable that in those cases where free hCG is low or not detected, it could be interesting to analyze the presence of LHCGR variants. Second, the proposed roles of hCG-H, hCGβ-H, and hCGβ through the TGF-β receptor in increasing TGCT aggressiveness should be better characterized, as blocking their interaction could lead to a better prognosis.

## 6. Conclusions

While hCG remains one of the most reliable serum markers for TGCT diagnosis and monitoring, its heterogenic expression among the different TGCT subtypes (i.e., not detectable in pure SE and some NST subtypes) underscores the need for more refined diagnostic tools. Emerging biomarkers, including miRNA-371a-3p and miRNA-375, may offer new promising alternatives to improve diagnostic accuracy; however, some efforts could be spent to increase the knowledge of the currently used markers, especially regarding hCG. About this, we must consider its variants and its post-translational modifications that could hold key prognostic value, despite a deeper validation being needed.

Finally, exploring LHCGR expression and its truncated forms may open new avenues for understanding tumor biology and therapeutic targeting. A comprehensive approach that combines classical serum markers with novel molecular indicators could significantly enhance early detection, prognostic precision, and personalized treatment strategies in TGCT patients. In order to promote the development of standardized approaches to take full advantage of emerging molecular biomarkers, the final aim is to incorporate them into international guidelines.

## Figures and Tables

**Table 1:** Summary of the main genetic and epigenetic alterations in TGCTs.

**Chromosomal**	**TGCT Type**	**Type of Alteration**
12p	SE and NSTs	Gain of chromosome 12p, i (12p)
11q	SE	Less frequent copies
2q, 8q, 8p, 10q, 15, 19q, 19p, 22	NSTs	Less frequent copies
**Somatic mutations/copy number gains**	**TGCT type**	**Type of alteration**
*KIT*	SE > NSTs	Activating mutations
*KRAS*	SE and NSTs	Activating mutations, copy number gain
*NRAS*	SE and NSTs	Activating mutations
*TP53*	SE and NSTs	Inactivating mutations
*MDM2*	SE and NSTs	Copy number gain
**DNA methylation status**	**TGCT type**	
Hypomethylation	SE	
Hypermethylation	NSTs	

**Table 2:** TNM classification for testicular cancer, reported by the latest European Association of Urology Guidelines on Testicular Cancer, 2025 edition [5]. ULN indicates the Upper Limit of Normal.

TNM Classification			
**pT—Primary Tumor**			
pTX	Primary tumor cannot be assessed		
pT0	No evidence of primary tumor		
pTis	Germ cell neoplasia in situ		
pT1	Tumor limited to testis (including rete testis) and epididymis without vascular/lymphatic invasion and without invasion of the epididymis		
pT2	Tumor limited to testis with vascular/lymphatic invasion, invading hilar soft tissue or the epididymis, or tumor extending through tunica albuginea with involvement of visceral tunica vaginalis		
pT3	Tumor invades spermatic cord with or without vascular/lymphatic invasion		
pT4	Tumor invades scrotum with or without vascular or lymphatic invasion		
**N—Regional Lymph Nodes—Clinical**			
NX	Regional lymph nodes cannot be assessed		
N0	No regional lymph node metastasis		
N1	Metastasis with a lymph node mass 2 cm or less in greatest dimensions or multiple lymph nodes, with no more than 2 cm in greatest dimension		
N2	Metastasis with a lymph node mass of more than 2 cm but no more than 5 cm in greatest dimension; more than 5 nodes positive, with no more than 5 cm; or evidence of extragonadal extension of tumor		
N3	Metastasis with a lymph node mass more than 5 cm in greatest dimension		
**Pn—Regional Lymph Nodes—Pathological**			
pNx	Regional lymph nodes cannot be assessed		
pNo	No regional lymph node metastasis		
pN1	Metastasis with a lymph node mass of 2 cm or less in greatest dimension and 5 or fewer positive nodes, with no more than 2 cm in greatest dimension		
pN2	Metastasis with a lymph node mass of more than 2 cm but no more than 5 cm in greatest dimension; or more than 5 nodes positive, with no more than 5 cm; or evidence of extranodal extension of tumour		
pN3	Metastasis with a lymph node mass more than 5 cm in greatest dimension		
**M—Distant metastasis**			
MX	Distant metastasis cannot be assessed		
M0	No distant metastasis		
M1	Distant metastasis		
	M1a	Non-regional lymph node(s) or lung metastasis	
	M1b	Distant metastasis other than non-regional lymph nodes and lung	
**S—Serum Tumor Markers (Pre-chemotherapy)**			
SX	Serum marker studies not available or not performed		
S0	Serum marker study levels within normal limits		
S1 S2 S3	**LDH (U/L)** <1.5 × ULN and 1.5–10 × ULN or >10 × ULN or	**hCG (mIU/mL)** <5000 and 5000–50,000 or >50,000 or	**AFP (ng/mL)** <1000 1000–10,000 >10,000

**Table 3:** Prognostic-based staging system for metastatic germ cell cancer by IGCCCG, reported in the European Association of Urology Guidelines on Testicular Cancer, 2025 edition [5].

**Good Prognosis Group**	
**Non-seminomas** 5-year PFS 90% 5-year OS 96%	All the following criteria: Testis/retro-peritoneal primary No non-pulmonary visceral metastases AFP < 1000 ng/mL hCGβ < 5000 IU/L (1000 ng/mL) LDH <1.5 × Upper Limit of Normal
**Seminoma** 5-year PFS 90% 5-year OS 96%	All the following criteria: Any primary site No non-pulmonary visceral metastases Normal AFP Any hCGβ Any LDH
**Intermediate prognosis group**	
**Non-seminomas** 5-year PFS 78% 5-year OS 89%	Any of the following criteria: Testis/retro-peritoneal primary No non-pulmonary visceral metastases AFP 1000–10,000 ng/mL or hCGβ 5000–50,000 IU/L or LDH 1.5–10 × Upper Limit of Normal
**Seminoma** 5-year PFS 79% 5-year OS 88%	All the following criteria: Any primary site No non-pulmonary visceral metastases Normal AFP Any hCGβ Any LDH
**Poor prognosis group**	
**Non-seminomas** 5-year PFS 78% 5-year OS 89%	Any of the following criteria: Mediastinal primary Non-pulmonary visceral metastasis AFP > 10,000 ng/mL or hCGβ > 50,000 IU/L (10,000 ng/mL) or LDH > 10 × Upper Limit of Normal
**Seminoma**	No patients classified
